# The Treatment of Acute Pneumonia

**Published:** 1907-04-06

**Authors:** Dyce Duckworth

**Affiliations:** Senior Physician to the Seamen's Hospital, Greenwich: Consulting Physician to St. Bartholomew's Hospital


### Hospital Clinics.

### THE TREATMENT OF ACUTE PNEUMONIA.

By SIR DYCE DUCKWORTH, M.D., LL.D., F.R.C.P., Senior Physician to the Seamen's Hospital, Greenwich: Consulting Physician to St. Bartholomew's Hospital.

A Lecture delivered in the London School of Tropical Medicine, Seamen's Hospital, Greenwich.

Gentlemen,—I wish at the outset of my remarks to-day to convey to you my opinion that the term pneumonia has now a wider significance than it had in the past. To recognise the disorder merely by the physical signs it causes in the lungs, and the presence of fever in the patient, is inadequate for a due conception of the malady.

We approach the subject now with very different conceptions from those which were held by our predecessors a quarter of a century ago. The progress of modern bacteriology has greatly transformed our knowledge, and made plain for us much that was formerly inexplicable. To our teachers of 30 years ago, pneumonia was practically regarded as a result of exposure to cold, and the mischief thus induced was supposed to fall upon the respiratory system alone. It was, indeed, regarded by some as a fever, somewhat specific, and at times it was observed to have a contagious or epidemic character. When the definite physical signs of its presence came to be made out accurately by the more modern methods of exploration, the idea of the local disturbance gained still firmer hold on the mind of most observers. The pneumonic process was watched as it began, proceeded, and terminated in death or recovery. The gross morbid anatomy was clearly described, and its histology, too, save in respect of the present toxic elements upon which we now lay so much stress. We have now learned that exposure to cold is not the sole factor in establishing pneumonia, although it is a highly predisposing one, and we find in the new edition of the "Nomenclature of Diseases," just issued, that this malady is placed in its epidemic form amongst the infective diseases. This, then, is the light in which we now have to regard pneumonia, and the specific agent which determines it in patients who are depressed, and rendered vulnerable by exposure or low health, is now fully revealed as the vicious pneumococcal microbe. This is a widely diffused organism, found in the mouths of healthy people, but then probably of little virulence. It is responsible for inflammatory mischief in many parts of the body, so that we meet with pleurisy, empyema, peritonitis, meningitis, arthritis, endo- and peri-carditis, nephritis, etc., rapidly acute disorders all dependent on this peccant element. Not seldom is it associated in its mischief with other virulent microbes such as staphylococci and streptococci (mixed infections). These considerations render our present conception of the pneumonic process very different from those formerly entertained, and in discussing treatment they have to be borne in mind. We meet with pneumonia as part of an influenzal process, of a rheumatic infection, and also as occurring in the course of enteric fever. We are not yet prepared for a classification of pneumonia which might be made as depending upon the several varieties of virulent bacilli which are either predominant or mixed together in the individual cases as the specific excitants of the disease.

I think it desirable to call your attention to a class of cases I have occasionally met with in boys. The disorder has followed a very prolonged stay [illegible] public swimming baths. Their bodies have th[illegible] become thoroughly chilled, and the possibilit[illegible] occurs to one of some special infection received at t[illegible] same time in the public bath, or of a lighting up [illegible] specific virulence of diplococcal and other microbe[illegible] already in the system, under the influence [illegible] depressing cold. These cases generally made favou[illegible]able progress.

The treatment of pneumonia has long furnishe[illegible] a famous battleground whereon physicians have m[illegible] in severe combat in the attempt to prove the speci[illegible] advantages of varying methods of therapeutics. [illegible] myself was brought up in the camp of a famo[illegible] general* who took the field with overweening co[illegible]fidence, and with supporting and restorative trea[illegible]ment declared that he won all along the line ov[illegible] his opponents who ventured to employ such dr[illegible] as antimony and mercury, or were so ignorant as [illegible] bleed in this disease. This battle is now fought o[illegible] and in the light of modern therapeutics we find th[illegible] while the truth was really on neither side, t[illegible] results of the restorative system stand out w[illegible] against those gained by what we must consider a[illegible] abuse of strong measures, but that in point of fa[illegible] there is no routine treatment for any person suffering from diseases that may be similar merely in name. Each case is, and must be, a study by itself, and while one may need to be treated by lowering measures, so-called, another may require supporting medication. As we shall find presently, we have to keep our armoury full of well-tried weapons, and must be ready to use any of them according to circumstances. Otherwise our hands will be tied by preconceptions, and we shall be theorists and not clinical physicians as we should always strive to be.

I will speak first about influenzal pneumonia. Influenza is now placed in the nomenclature of the Royal College of Physicians amongst the infective diseases. When the lungs are involved we may f[illegible] three several conditions of pulmonic inflamma[illegible] First, we find ordinary croupous or lobar [illegible]monia. Secondly, an engorgement of the [illegible] without actual consolidation, a condition desc[illegible] by Sir Andrew Clark as "soaked" lung. Thi[illegible] we meet with broncho-pneumonia or the so-c[illegible] catarrhal form, one rarely occurring in ad[illegible]

* When House Physician to the late Professor Hug[illegible] Bennett of Edinburgh.

ough frequent in children and old people. The 'sical signs accompanying the second condition ‚ve named are peculiar. No bronchial breathing audible over the patches of dullness; there are 'r silent areas and others over which mucous ‚ies may be heard. In the influenzal variety of broncho-pneumonia patches of dullness may be 'cund, and there may be migratory pneumonia with limited bronchial breathing and more abundant mucous râles.

Some of the worst cases fall into the second category where the lungs are soaked. Asthenia is the prevailing character in this case. Many of the fatal cases in elderly persons are of this character, the cardiac failure being very marked. In the more classical forms of pneumonia it is common, as you e aware, to find the bases rather than other por- ons of the lungs involved. The right base is especi- ly obnoxious to the disorder, and so far as 'ognosis in any case is concerned this is rather of vourable import than otherwise. Pneumonia of e apex is peculiar in several respects. In this case e is apt to be anxious as to the future, because is condition may clear up tardily and prove a dus for the onset of tuberculosis.

Again, it is very singular to find how powerfully ıpressed the nervous system may be in apex- ıeumonia. Delirium is extremely frequent in such ses, both in young and elderly patients. This is much recognised by me that I generally warn the ‚endants early of the possibility of this symptom pervening. An outbreak of herpes about the ›uth is of some significance. I take it on the whole be a sign of favourable import, and prefer to see Certainly some of the worst cases never throw herpes, but it is not always a prognostic for od. Some physicians speak of herpetic pneumonia a distinct form, generally likely to do well. The aracters of the expectoration you will not always d to accord with that commonly given in text- ›oks. Rusty sputa are a good indication, and if ıney are very bloody you are not to think ill of the case on that account. A sparely-built man suffering from acute pneumonia with very bloody sputa is likely to do well, though you might be alarmed at such a conjunction. In many cases you will see no rusty sputa, and there may be no expectoration of a'y kind. In rheumatic pneumonia it is the rule to have either mucous or muco-purulent expectoration without any blood. Of somewhat worse import is the dark-coloured sputum which has been likened to "prune juice" or "liquorice water," and which may become of a dingy green colour. This is an indication of a low type of the disease, indicative of asthenia with a tendency to gangrene. Cases illustrating this are apt to sink, but you must not ays expect an untoward issue in any given in- 'e on this account. We sometimes meet with putum in the persons of drunkards affected pneumonia. You know that a drunkard is a ;ularly bad subject for the disease, and if both are involved he will almost certainly die of it. far I have rather discussed some prognostic ts in reference to pneumonia. I must add more. has been taught that an acute pneumonia occur- g in a previously healthy young man should pass off favourably and leave no harm behind it. This is true. But a person who has had one attack is rather more likely than another to have a second one in the course of his life. This must either indicate some special diathetic vulnerability or suggest to us that one attack somewhat predisposes to a second. Recurring attacks are not uncommon in gouty persons. Persons suffering from pulmonary emphysema are particularly bad subjects for pneumonia. They almost always die of it. Hence it is a fatal disease for those who have long had bronchitis. Any renal disorder is a serious factor in a given case. Cases of chronic parenchymatous nephritis are almost as certainly carried off by pneumonia, as are persons over 50 years of age by typhus fever. Renal integrity or adequacy is necessary to the effective metabolism which is called for in any acute disease. In some cases in elderly patients we are justified in believing that the kidneys are hardly fully efficient, inasmuch as some degree of interstitial nephritis or cirrhosis may be present, and this is a grave complication. A measure of leucocytosis, if discovered, is of favourable omen. A considerable degree of pyrexia is to be expected, and this appears to be necessary for recovery. A temperature over 104° may, however, excite some apprehension. From the fifth to the eighth day we look for a crisis with a rapid fall of temperature and sweating. With this comes a good deal of general relief, but, withal, no alteration in the physical condition of the lungs. Indeed, the classical signs in the lungs may only be developing as the pyrexia ceases.

For many days after a favourable crisis you will find the pneumonic signs well marked, though steadily subsiding. Patients with pneumonia generally come first under our observation with marked fever and lassitude. The face is flushed, the eyes are bright, and the lips dry. There is often frontal headache. The respirations are hurried, about half the pulse-rate. There is a short teasing cough. We place them in a warm bed. (Sir William Gull was wont to regard this as the all-essential part of the treatment.) If there is pain in the chest, a warm linseed poultice—the so-called jacket poultice—is of great value. I am not sure that this treatment is necessary or even desirable in warm weather unless there is pain. Of ice poultices I have no experience. I should dislike them myself if I had pneumonia, and I am quite satisfied with the older method. The pain is almost certainly due to some degree of pleuritis over the engorged lung.

Practice varies in respect of early internal medication. I have for long been best satisfied in most cases with quinine given in two or three grain doses every four or six hours in a mixture with chloroform water. Another good remedy at this stage is potassium bicarbonate with ammonium carbonate, 15 grains of the former and four or five of the latter four times a day. Ammonium chloride, well sweetened, is also of much value in 15 grain doses every six hours. I seldom bleed in pneumonia. The indication for it is extreme engorgement of the right side of the heart and of the venous system. In this case a small bleeding from the arm to the extent of six or eight ounces may be very helpful and bring relief. The idea of cutting short the disease by bleeding is not now entertained as it was formerly. When the lung is consolidated such a result cannot be looked for. But I can well understand that a free bleeding within 24 hours of the onset of the disease may materially check and circumscribe its subsequent development. We seldom have such an opportunity for this interference. In aged or broken-down subjects you will hesitate to draw blood unless for mechanical relief, and then only to the extent of a few ounces. A slight degree of jaundice may occur in patients with pneumonia. This was formerly thought to arise only in cases where the lower lobe of the right lung was involved. Most pneumonias occur in this situation. It is now known to be due to engorgement of the liver due to a loaded state of the right chambers of the heart, and with this is commonly found a turgid state of the jugular veins. The indication here is to stimulate the circulation, and reinforce cardiac power by digitalis, strychnine, and alcohol. A mild mercurial purgative may aid in this condition. Failing these measures, a small bleeding may be practised with advantage.

I have said nothing respecting the use of antimony. I have not employed it for many years. I think it may be helpful in small doses, with alkalies, during the first few days in young subjects with good cardiac power and a tense pulse, but it cannot long be borne with advantage, and the treatment must be changed. The so-called heroic treatment was supposed to cause a larger mortality than that now practised and called the "supporting" plan. Dr. Loomis, of New York, has called attention to some statistics collected in Boston which throw light on this matter, and the conclusion to be drawn from these is that the mortality from pneumonia varies very little under any method of treatment. But, as you know, anything can be proved, and any cause be supported, by an appeal to statistics. I am not much enamoured of them in medical matters. By the light of modern bacteriology we are led to consider the question of serum therapeutics in pneumonia. It is known that cultures of pneumococci can be obtained from the patient's blood during the progress of the disease, but so far there are no satisfactory results, so far as I can learn, from the use of anti-pneumococcal serum. We are, however, entitled to look for benefits in this therapeutic direction in the near future, and must hope for a satisfactory establishment of them. The symptoms of restlessness, distress of breathing, and insomnia are commonly witnessed, and they compel us to consider the propriety of using sedatives and more particularly opiates. There has long been a general indisposition to employ opium in any form in the course of pneumonia, owing to fear of its checking secretion and expectoration, and rendering the respiratory centre indifferent to centripetal impressions.

These objections do not apply to a proper and appropriate use of this drug, and a large experience of its use enables it to be affirmed that it proves most helpful in procuring quietude and sleep. It is best given in the form of morphine together with the compound spirit of ether (Hoffmann's anodyne). Fifteen minims of the liq. morphinæ hydrochloridi may be prescribed with a drachm of Hoffmann's anodyne in an ounce of chloroform-water. Another effectual hypnotic is 5 grains of musk rubbed up with half an ounce of mucilage of acacia, a drachm of Hoffmann's anodyne with sufficient chloroform water. These may be repeated in two hours if necessary. The dietetic treatment is that for acute disease with pyrexia. Milk diluted with barley-water or some aërated water is the staple food, and given with ice is very grateful. Beef-tea, veal, mutton, and chicken-broths are all excellent. If there is dislike to any of these, strong beef-essence given i nteaspoonful doses in a wineglassful of iced water may be readily taken. Whey is a very good liquid form of nutriment. Eggs and milk, or egg-flip are also useful. Draughts of Vichy or of plain cold water, two ounces at a time, at intervals, should not be forgotten.

The latter brings me to discuss the question of alcoholic stimulation in pneumonia. Our practice here is guided by the condition of the circulation, the life-history and age of the patient, and the degree of pyrexia. Alcohol must be prescribed or withheld in disease as any other remedial agent should be. Alcohol is not generally required in any case for the first few days. In young persons the disease may run its course without its aid at any time. A soft bounding pulse, increasing temperature, cyanosis, and accompanying weakness of the heart's sounds call for it. We give port wine or brandy, or both, in quantities varying according to circumstances from one to eight ounces, but in broken-down patients, drunkards, and old persons more may be required. I often add digitalis in the form of 10 or 12 minims of the tincture to each dose of the medicine, and with this as much tincture of nux vomica. With any obvious failure of cardiac power, I employ hypodermic injections of strychnine, 2 minims of the liquor with 10 of pure ether, and this is repeated hourly if necessary. I specially call your attention to this latter practice. I have certainly known it save life and turn the scale in a right direction at precarious moments. Musk is a remedy of great power in such a condition. It may be given in pill, in doses of 2 to 10 grains in emulsion, or in the form of a tincture added to the medicine. It must be of good quality, and the only objection to its use is its cost. Inhalation of warmed oxygen gas is certainly useful in severe cases. It should be employed early in the case, and before cyanosis supervenes. So soon as the patient has surpassed the crisis and craves for solid food, he may have it. The amount of stimulants may be reduced day by day according to circumstances. Convalescence is commonly rapid in the young and in previously healthy persons. The physical signs in the lungs may only gradually disappear, and sometimes they subside very slowly. Resolution may in such cases be hastened by the application of a cantharidine blister over the affected side. Tardy resolution is a source of anxiety to us in persons of tubercular habit, and especially is this the case if, as I have remarked, there has been an apex-pneumonia. Again, delayed resolution may be associated with a progressive empyema, interlobar or general.

In these persons the greatest care must be taken to procure as rapid and complete a recovery as possible. Change of climate and special precautions during the succeeding winter are generally imperative. Woollen underclothing should always be worn by everybody. It is not unnecessary to tell you this. You would be surprised to know how many persons, apparently intelligent, neglect this simple preventive of pulmonary and abdominal disorders. In no part of the world may this simple precaution be neglected, and it is as necessary in the tropics as in higher latitudes. Overheated rooms in winter are, I believe, a fertile source of pneumonia, and I am disposed thus to account for the frequency and perilous nature of the pneumonia which is so common in winter on the continent of Europe and in American cities. In that country and on the continent of Europe the houses are warmed by stoves, and the windows are rarely opened. The atmosphere is thus dry and unwholesome, and it becomes positively dangerous under these conditions to go into the open air during a frost unless wrapped in fur. Railway-travelling in America and on the European continent in winter is particularly dangerous owing to the improper overheating and bad ventilation of the carriages. The sudden change of temperature is very risky for any but the most robust. In this country with open fireplaces (and less genial warmth, no doubt) in our rooms and passages, we escape this peril to a large extent. But with us pneumonia is not essentially a disease of winter. Bronchitis is far more common. Spring and early summer bring us most cases of pneumonia. People are induced by lengthening days to discard winter clothing, and to believe that summer is upon them, and, therefore, to expose themselves to dangerous chills. We seldom lose east winds before June, and winter precautions are, therefore, to be taken up to that time, even at the risk of a little discomfort.

Lastly, bear well in mind this point—that the chief danger in any case of pneumonia concerns the heart rather than the lungs. The pulmonary circulation, being gravely impeded, throws undue work on the myocardium, especially upon the right chambers of the heart. Distension and strain may prove too much for their capacity and propulsion. Hence the special risks attaching to double pneumonia, to the co-existence of pulmonary emphysema, and the impaired nutrition of the myocardium in elderly patients.

The condition of the circulation has, therefore, to be specially noted and treated from the outset of any case. Care must be taken not to overload the stomach with excess of fluid, and to avoid flatulent distension of the bowels. Half a dozen leeches applied over the epigastrium may prove very helpful in relieving the right side of the heart. It is well to be timely in all measures for averting cardiac failure. Not seldom efforts in this direction are deferred till it is too late to secure benefit from them. The room should be well ventilated, kept at a temperature of 65° F. and free from draughts. The bed should be away from the wall, and no heavy bedclothes or duvets should be permitted to cover the patient. One often finds these patients swathed in excess of blankets with a pyrexia of 103° or more, unable to express their discomfort and suffering caused by well-meant but inappropriate management. Be assured that successful clinical management of all acute diseases is largely dependent on attention to details which demand close observation on the part of the practitioner, and may be neglected at the peril of the patient.

